# Transcatheter vs. surgical aortic valve replacement in bicuspid aortic valve stenosis

**DOI:** 10.1093/ehjopen/oeaf110

**Published:** 2025-08-29

**Authors:** Joseph Kassab, Parth Desai, Neil Keshvani, Katy Lonergan, Amit Goyal, Ambarish Pandey, Saket Girotra, Dharam J Kumbhani

**Affiliations:** Division of Cardiovascular Medicine, Department of Internal Medicine, UT Southwestern Medical Center, 2001 Inwood Road Suite WC05.870, Dallas, TX 75390-9254, USA; Division of Cardiovascular Medicine, Department of Internal Medicine, UT Southwestern Medical Center, 2001 Inwood Road Suite WC05.870, Dallas, TX 75390-9254, USA; Division of Cardiovascular Medicine, Department of Internal Medicine, UT Southwestern Medical Center, 2001 Inwood Road Suite WC05.870, Dallas, TX 75390-9254, USA; Division of Cardiovascular Medicine, Department of Internal Medicine, UT Southwestern Medical Center, 2001 Inwood Road Suite WC05.870, Dallas, TX 75390-9254, USA; Division of Cardiovascular Medicine, Department of Internal Medicine, UT Southwestern Medical Center, 2001 Inwood Road Suite WC05.870, Dallas, TX 75390-9254, USA; Division of Cardiovascular Medicine, Department of Internal Medicine, UT Southwestern Medical Center, 2001 Inwood Road Suite WC05.870, Dallas, TX 75390-9254, USA; Division of Cardiovascular Medicine, Department of Internal Medicine, UT Southwestern Medical Center, 2001 Inwood Road Suite WC05.870, Dallas, TX 75390-9254, USA; Division of Cardiovascular Medicine, Department of Internal Medicine, UT Southwestern Medical Center, 2001 Inwood Road Suite WC05.870, Dallas, TX 75390-9254, USA

**Keywords:** Bicuspid Aortic Valve Stenosis, TAVR, SAVR

## Abstract

**Aims:**

Patients with bicuspid aortic valve (BAV) stenosis were excluded from major TAVR trials, and data comparing TAVR and SAVR in this population remain limited. To compare real-world, risk-adjusted outcomes of TAVR vs. SAVR in patients with BAV stenosis.

**Methods and results:**

We conducted a retrospective cohort analysis using the TriNetX research network database. Adults (≥18 years) with echocardiographically confirmed BAV stenosis undergoing isolated TAVR or SAVR from 2012 to 2022 were included. Patients with prior cardiac procedures or concomitant cardiac interventions were excluded. Propensity score matching (PSM) (1:1) was used to balance covariates. Primary outcomes were 2-year all-cause mortality, stroke, and valve re-intervention. Secondary outcomes included new pacemaker implantation (PPM), 30-day AKI, and bleeding. 5547 patients (TAVR: 1444; SAVR: 4103) were included. In unadjusted analysis, TAVR patients were sicker and older at baseline and had a higher risk of death and/or stroke compared with those who underwent SAVR (10.9% vs. 5.37%, *P* < 0.0001). Following PSM, 663 matched pairs were analyzed with all covariates balanced. At 2 years, all-cause mortality (TAVR: 4.8% vs. SAVR: 5.3%; OR: 0.91, *P* = 0.71) and stroke (TAVR: 7.3% vs. SAVR: 4.5%; OR: 1.67, *P* = 0.058) were similar between the two groups. Re-intervention rates were low and comparable. TAVR was associated with higher PPM rates but lower AKI and bleeding rates.

**Conclusion:**

In propensity-matched BAV patients, TAVR and SAVR demonstrated comparable 2-year mortality, stroke, and re-intervention rates. These findings support TAVR as a viable option in appropriately selected BAV patients, warranting further prospective validation.

## Graphical Abstract

**Graphical Abstract:**
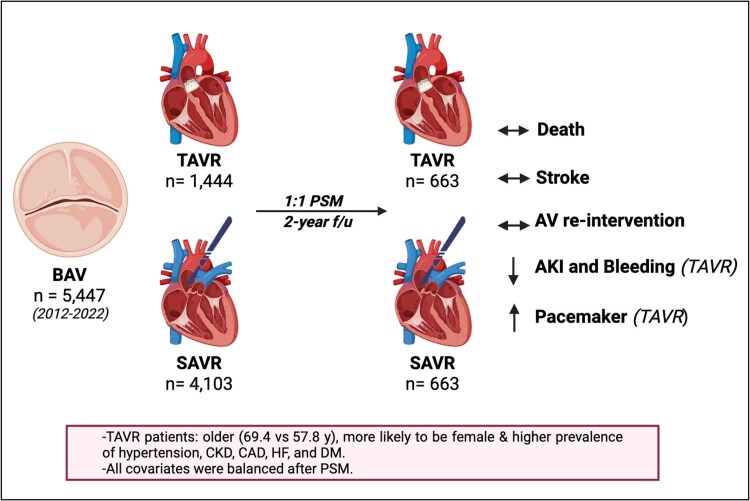

## Introduction

Early pivotal transcatheter aortic valve replacement (TAVR) trials excluded patients with bicuspid aortic valve (BAV) stenosis.^1–3^ However, evidence suggests that TAVR in this population is associated with no difference in 30-day and 1-year mortality compared with trileaflet aortic valves.^4^ Large-scale studies comparing surgical aortic valve replacement (SAVR) and TAVR in BAV stenosis are still lacking, and current guidelines recommend that TAVR may be considered as an alternative to SAVR only after careful evaluation.^5^ The purpose of the current study was to evaluate real-world contemporary outcomes of TAVR vs. SAVR in patients with BAV stenosis. We hypothesized that TAVR and SAVR would be associated with a similar risk of mortality and/or stroke at 2 years in patients with comparable baseline characteristics.

## Methods

We conducted a retrospective cohort analysis of de-identified, aggregate patient data from the TriNetX research network, which contains data from the electronic health records of ∼115 million patients from 72 healthcare organizations, primarily in the United States. Patients aged ≥ 18 years old with TTE-confirmed BAV stenosis who underwent AVR between January 2012 and January 2022 were identified. Patients with concomitant aortic procedures, coronary procedures, valvular procedures, prior heart surgery or valve procedure, pure aortic regurgitation, mechanical support, endocarditis, cardiogenic shock, or emergency procedures were excluded. Included patients were divided into two groups based on whether they underwent isolated TAVR or isolated SAVR. Primary and secondary outcomes were analyzed over a 2-year follow-up period. The primary outcomes of interest included all-cause mortality and stroke, as well as risk of re-intervention. Secondary outcomes included permanent pacemaker (PPM) implantation, 30-day acute kidney injury (AKI), and procedure-related hemorrhage (with or without transfusion). Covariates (including baseline demographics, prescribed medications, comorbidities, LVEF, and hemoglobin, serum creatinine, and eGFR on day of procedure) were matched extensively by 1:1 PSM using the greedy nearest-neighbor algorithm with a caliper of 0.1 pooled standardized mean differences (SMD). Any characteristic with a SMD between cohorts lower than 0.1 was considered well-matched. The measures of association included odds ratios (ORs) on the matched population for primary and secondary outcomes. Survival analyses were performed for each outcome by plotting Kaplan-Meier curves with log-rank tests; additionally, Cox proportional hazard models were used to calculate the HR to compare the two groups. All hazard ratios were estimated in the propensity score-matched cohort, ensuring covariate balance between groups. Death was considered a censoring event. A value of *P*  *<* 0.05 was considered statistically significant. Analyses were conducted using the embedded R model within the TriNetX platform. The study was exempt from institutional review board/ethics committee approval as it leveraged a de-identified database. Data were analyzed and interpreted by the authors. All authors reviewed the manuscript and afﬁrmed the accuracy and completeness of the data.

## Results

**Figure 1:**
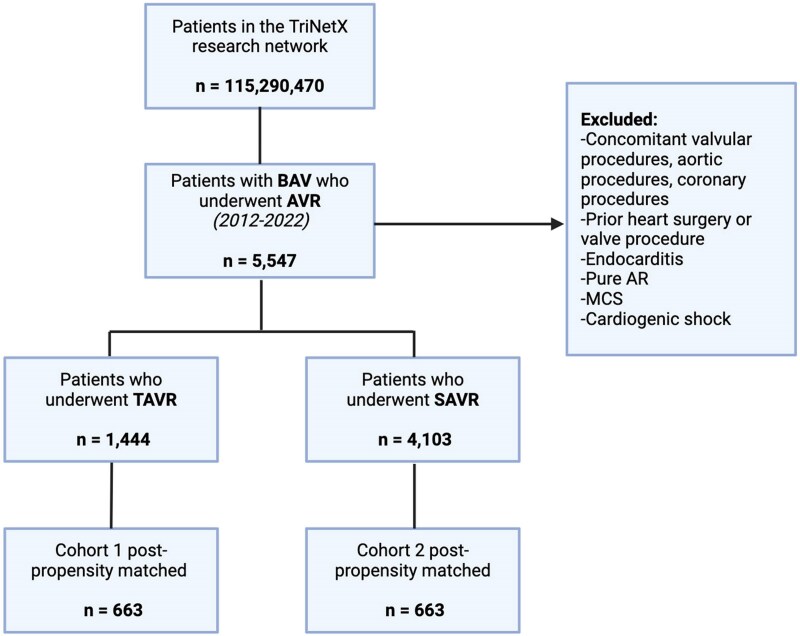
Study flow chart. AVR, aortic valve replacement; BAV, bicuspid aortic valve; SAVR, surgical aortic valve replacement; TAVR, transcatheter aortic valve replacement.

**Figure 2:**
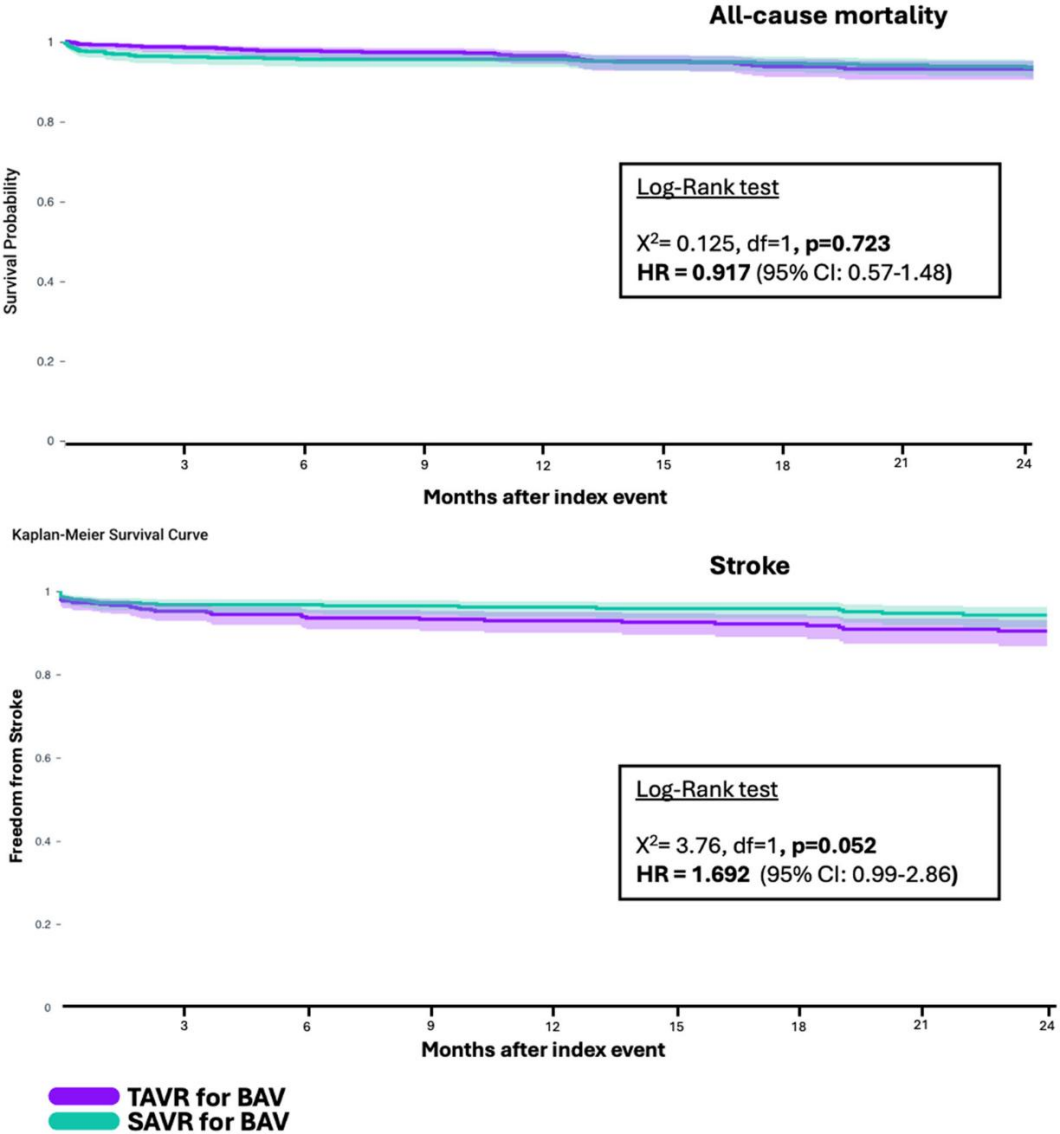
Time-to-event analysis of primary outcomes. Kaplan-Meier survival curves comparing 2-year outcomes of all-cause mortality (top panel) and stroke (bottom panel) between patients with BAV stenosis treated with TAVR vs. SAVR. No significant differences were found in mortality or stroke between the two cohorts after robust propensity score matching. All hazard ratios were estimated in the propensity score-matched cohort, ensuring covariate balance between groups. BAV, bicuspid aortic valve; HR, Hazard ratio; SAVR, surgical aortic valve replacement; TAVR, transcatheter aortic valve replacement.

Out of 5547 patients with BAV stenosis met inclusion criteria. Of these, 1444 patients underwent TAVR and 4103 underwent SAVR (92% receiving a bioprosthetic valve). After PSM, 663 matched patients remained in each cohort and were included in this analysis. *Figure 1* highlights the study’s flowchart. Select baseline patient characteristics are showcased in *Table 1*. In the unmatched cohort, patients treated who underwent TAVR were older (69.4 vs. 57.8 years; *P*  *<* 0.05) and more likely to be female (37.2% vs. 25.6%; *P*  *<* 0.05) compared with those who underwent SAVR. Furthermore, patients who underwent TAVR had a higher prevalence of hypertension, chronic kidney disease, coronary artery disease, heart failure, and diabetes mellitus. In the unadjusted analysis, patients who underwent TAVR had a higher risk of death and/or stroke compared with those who underwent SAVR (10.9% vs. 5.37%, *P* < 0.0001). Following tight PSM, baseline characteristics of the two groups were similar (*n* = 663 per group; mean age: 64.7 years; 31% female; 84% white; mean LVEF: 54.9%). No residual imbalances were found (SD: < 0.1 for all covariates, *Table 1*). The median follow-up duration was 615 days (IQR: 580 days) for patients in the TAVR group and 679 days (IQR: 589 days) for those in the SAVR group. All-cause mortality did not differ significantly between the two groups [TAVR: 4.8% vs. SAVR: 5.3%; OR: 0.91 (95% CI: 0.56–1.49); *P* = 0.71]. The incidence of stroke also did not differ significantly [TAVR: 7.3% vs. SAVR: 4.5%; OR: 1.67 (95% CI: 0.98–2.85); *P* = 0.058]. Aortic re-intervention rates were similar between the two cohorts [TAVR: 0.32% vs. SAVR: 0.28%; OR: 1.17 (95% CI: 0.81–1.69); *P* = 0.41]. PPM implantation was significantly more frequent in the TAVR group [11.8% vs. 8.1%; OR: 1.504 (95% CI: 1.04–2.17); *P* = 0.028]. AKI and bleeding at 30 days were less common following TAVR [OR: 0.376 (95% CI: 0.23–0.60); *P* < 0.0001 and OR: 0.23 (95% CI: 0.12–0.43); *P* < 0.0001, respectively]. Time-to-event analysis of the primary outcomes, shown in *Figure 2*, also demonstrated a similar risk of death [matched HR = 0.917 (95% CI: 0.57–1.48); *P* = 0.723] and risk of stroke [matched HR = 1.692 (95% CI: 0.998–2.862); *P* = 0.052] between TAVR and SAVR.

**Table 1:** Baseline characteristics of the study cohort before and after propensity score matching

Characteristic	TAVR (*N* = 1444)	SAVR (*N* = 4103)	SMD (Pre-match)	TAVR (*N* = 663)	SAVR (*N* = 663)	SMD (Post-match)
**Demographics**						
Age at index (years)	69.6 ± 10.7	57.7 ± 12.6	1.02	64.8 ± 11.6	64.5 ± 9.9	0.02
Female (%)	541 (37.5)	1070 (26.1)	0.25	218 (32.9)	200 (30.3)	0.05
White (%)	1215 (84.2)	3540 (86.3)	0.06	564 (85.1)	553 (83.5)	0.04
Hispanic (%)	82 (5.7)	242 (5.9)	0.04	37 (5.7)	38 (5.8)	0.03
**Comorbidities**						
Hypertension (%)	1215 (84.2)	3110 (75.8)	0.21	543 (82.0)	524 (79.1)	0.07
Hyperlipidemia (%)	974 (67.5)	2359 (57.5)	0.25	456 (68.8)	462 (69.8)	0.01
Heart failure (%)	940 (65.1)	1563 (38.1)	0.65	387 (58.5)	383 (57.8)	0.01
Diabetes (%)	469 (32.5)	960 (23.4)	0.21	240 (36.3)	239 (36.1)	0.003
Atrial fibrillation/flutter (%)	293 (20.3)	681 (16.6)	0.09	135 (20.5)	137 (20.7)	0.004
Coronary artery disease (%)	885 (61.3)	1870 (45.6)	0.33	379 (57.2)	383 (57.9)	0.01
Chronic kidney disease (%)	401 (27.8)	521 (12.7)	0.38	137 (20.7)	155 (23.5)	0.06
Obesity (%)	447 (31.0)	1259 (30.7)	0.007	231 (34.9)	226 (34.2)	0.01
Chronic lung disease (%)	349 (24.2)	586 (14.3)	0.25	127 (19.3)	141 (21.4)	0.05
Cancer (%)	129 (9.0)	369 (9.0)	0.01	59 (9.0)	59 (9.0)	0.01
Cerebrovascular accident (%)	95 (6.6)	237 (5.8)	0.03	39 (6.0)	48 (7.3)	0.05
Peripheral artery disease (%)	145 (10.1)	229 (5.6)	0.17	48 (7.3)	53 (8.0)	0.03
Tobacco use (%)	202 (14.0)	607 (14.8)	0.02	97 (14.7)	95 (14.4)	0.01
Alcohol use (%)	209 (14.5)	648 (15.8)	0.06	94 (14.2)	100 (15.1)	0.04
**Metrics**						
LVEF (%)	57.8 ± 14.1	57.6 ± 11.2	0.01	54.7 ± 14.2	55.0 ± 12.8	0.03
HbA1c (%)	6.17 ± 1.12	5.84 ± 1.08	0.29	6.21 ± 1.28	6.19 ± 1.19	0.022
Hemoglobin (g/dL)	12.2 ± 2.28	13.5 ± 2.50	0.03	12.4 ± 2.43	12.9 ± 2.60	0.02
Serum creatinine (mg/dL)	1.1 ± 0.96	0.93 ± 0.53	0.21	1.11 ± 1.15	0.98 ± 0.48	0.09

SAVR, Surgical Aortic Valve Replacement; SMD, Standardized Mean Difference; TAVR, Transcatheter Aortic Valve Replacement.

## Discussion

Over the last decade, TAVR has emerged as a valid alternative to SAVR in elderly patients with severe aortic stenosis. Recent studies have further expanded TAVR indications to include younger, lower-risk patient populations.^3^ To the best of our knowledge, our study is among the few real-world studies assessing the efficacy of TAVR specifically in a large sample of patients with BAV stenosis. We found that compared with SAVR, TAVR is associated with similar rates of all-cause mortality and stroke at 2 years as well as similar rates of re-intervention. A recent meta-analysis examining short-term outcomes (in-hospital and 30-day) similarly demonstrated comparable outcomes between TAVR and SAVR.^6^ Our study strengthens and expands the existing literature by evaluating longer-term outcomes. Recent data from the NOTION-2 trial, which compared TAVR to SAVR in younger, lower surgical risk patients with severe tricuspid or bicuspid AS, highlighted a higher composite end point of all-cause mortality, stroke, or rehospitalization in BAV patients treated with TAVR compared with SAVR. However, the association was not statistically significant (HR 3.8; 95% CI: 0.8–18.5).^7^ Additionally, a recently published analysis of Medicare outcomes comparing TAVR and SAVR in patients with BAV reported worse long-term outcomes, notably all-cause mortality and stroke, among those who underwent TAVR compared with SAVR.^8^ While these findings may appear to contradict our results, it is worth noting that our unadjusted analysis also showed a higher risk of mortality and stroke in the TAVR group, likely reflecting a greater burden of comorbidities and baseline frailty, as demonstrated in the pre-PSM characteristics, hence introducing a confounding by indication bias. We tentatively tried to decrease the risk of confounding by robustly matching the two cohorts. Nonetheless, differences in baseline population profiles (frailty, registry used) may explain the discrepancy in findings. Moreover, it is worth noting that in our propensity-matched analysis, the difference in stroke rates between TAVR and SAVR also demonstrated a trend toward significance (*P* = 0.058), raising the possibility of a higher risk of stroke with TAVR in this population. Nonetheless, we believe our results support the consideration of TAVR as a viable option for appropriately selected patients with BAV stenosis, particularly when guided by a multidisciplinary Heart Team. Together with the current body of literature, our study further highlights the need for prospective, adequately powered trials specifically comparing TAVR and SAVR in patients with BAV.

### Limitations

Data for this study were obtained from an aggregate electronic health record database (TriNetX). As such, the accuracy of reported health conditions and the completeness of captured outcomes occurring outside of the database may be limited. Additionally, Society of Thoracic Surgeons (STS) risk scores were unavailable for analysis and direct adjustment. However, we attempted to mitigate this limitation by including all variables used in the STS operative risk calculator within our matched propensity score model. Despite robust propensity matching, potential selection bias from unmeasured confounding factors remains a limitation. Moreover, we acknowledge the lack of detailed information on specific valve types or prosthesis models in the TriNetX database as well as BAV subtypes (e.g. Sievers classification). Nevertheless, we believe this study provides valuable insights into the comparative outcomes of TAVR vs. SAVR in patients with BAV stenosis.

## Lead author biography

Dharam Kumbhani MD is the Section Chief of Interventional Cardiology and a Professor of Medicine in UT Southwestern. He is also the Director of the Cardiac Catheterization Laboratory at Clements University Hospital. He holds the Jim and Norma Smith Distinguished Chair for Interventional Cardiology.
